# Chronic Exposure to Diquat Causes Reproductive Toxicity in Female Mice

**DOI:** 10.1371/journal.pone.0147075

**Published:** 2016-01-19

**Authors:** Jia-Qing Zhang, Bin-Wen Gao, Jing Wang, Xian-Wei Wang, Qiao-Ling Ren, Jun-Feng Chen, Qiang Ma, Bao-song Xing

**Affiliations:** 1 Institute of Animal Husbandry and Veterinary Science, Henan Academy of Agricultural Sciences, Zhengzhou, China; 2 Henan Provincial Animal Husbandry General Station, Zhengzhou, China; China Agricultural University, CHINA

## Abstract

Diquat is a bipyridyl herbicide that has been widely used as a model chemical for in vivo studies of oxidative stress due to its generation of superoxide anions, and cytotoxic effects. There is little information regarding the toxic effects of diquat on the female reproductive system, particularly ovarian function. Thus, we investigated the reproductive toxic effects of diquat on female mice. Chronic exposure to diquat reduced ovary weights, induced ovarian oxidative stress, resulted in granulosa cell apoptosis, and disrupted oocyte developmental competence, as shown by reactive oxygen species (ROS) accumulation, decreased polar body extrusion rates and increased apoptosis-related genes expression. Additionally, after diquat treatment, the numbers of fetal mice and litter sizes were significantly reduced compared to those of control mice. Thus, our results indicated that chronic exposure to diquat induced reproductive toxicity in female mice by promoting the ROS production of gruanousa cells and ooctyes, impairing follicle development, inducing apoptosis, and reducing oocyte quality. In conclusion, our findings indicate that diquat can be used as a potent and efficient chemical for in vivo studies of female reproductive toxicity induced by oxidative stress. Moreover, the findings from this study will further enlarge imitative research investigating the effect of ovarian damage induced by oxidative stress on reproductive performance and possible mechanisms of action in large domestic animals.

## Introduction

Diquat (1,1′-ethylene-2,2′-bipyridilium) is a bipyridyl herbicide for the control of aquatic weeds and broad-leaved weeds among fruit, vegetables, and as a preharvest desiccant for seed and fodder crops such as rice and sunflower[1]. Superoxide anion radical generation and subsequently hydrogen peroxide production are thought to be key cytotoxic mechanisms induced by diquat [2]. In rats and mice, high dose injection with diquat causes liver necrosis, lung injury, and animal death due to acute oxidative stress generation [3–5]. It is reported that diquat exposure caused 10–40 times higher ROS production compared to paraquat [6]. Weaned pigs treated with diquat can cause obvious reduction in activities of antioxidant enzymes and enhancement in malondialdehyde (MDA) concentration in the plasma and liver [7–9]. Exposure to formulations containing diquat alone as well as in combination with other herbicidal agents has been shown to induce toxicity[10]. Other studies have also indicated that diquat could cause severe and extensive mucosal damage, including stomach, esophagus, mouth, and small intestine[1]. Furthermore, diquat is also widely used as a pro-oxidant to induce oxidative stress in different animal models [9, 11, 12].

The ovary is an important organ in female reproductive system, as it generates oocytes and regulates hormones secretion[13]. In mammalian ovary, follicles are very important functional units, as they contain the oocytes for ovulation and fertilization[14]. It is reported that toxic damage to ovarian follicles may cause blocked ovulation, which in turn may lead to infertility[14]. Reactive oxygen species (ROS) and antioxidants remain in balance in a well-developed follicle under both internal and external factors[15]. Oxidative stress occurs when formation of ROS exceeds the ability of the cells to defend themselves from increased ROS[16]. Follicular granulosa cells play an important function in various stages of follicle development, ovulation, and oocyte maturation. Furthermore, Studies have shown that follicular atresia is mainly due to granulsoa cell apoptosis, and that oxidative stress-induced apoptosis is believed an important cause for atresia[17, 18]. Excessive ROS levels in granulosa cells can reduce oocyte quality and subsequently early embryos development competence[19]. Oocytes and early embryos are extremely sensitive to excessive ROS levels, which reduces oocyte quality, affects embryo development and results in early embryo fragmentation[20]. It was also reported that excessive ROS levels could damage important molecules and structures in oocytes and embryos, and accelerates their aging and death[21, 22].

However, there is little information regarding the effect of diquat on the female reproductive system, particularly ovarian function. Thus, we designed the present work to assess the reproductive toxic effects of diquat in female mice.

## Materials and Methods

### Ethics statement

The experimental procedures followed the actual law of animal protection that was approved by the Animal Care Advisory Committee of Henan Province, China. Ovarian isolations were performed under anesthesia as described below. Throughout the course of this study, all efforts were made to minimize animal suffering.

### Animals and Chemicals

Female ICR mice were obtained from Experimental Animal Center of Henan Province, China. The animals were housed in plastic cages in a room kept under standardized conditions at a temperature of 24±2°C, 20% humidity, and a 12-h light/dark cycle, with free access to tap water and food throughout the study. Diquat was purchased from Sigma (St Louis, MO). Kits for testing superoxide dismutase (SOD), catalase (CAT) and glutathione peroxidase (GPx) activities and MDA were purchased from Nanjing Jiancheng Biotechnology Institute, China. Intracellular ROS red fluorescence determination kit was purchased from GENMED (Shanghai, China). The in situ cell death fluorescein detection kit was obtained from Roche (Mannheim, Germany). All other chemicals were of analytical reagent grade.

### Experiment Design and Treatments

Ninety healthy female ICR mice (beginning at 21 days old) were divided randomly into three groups of 30 mice each. Group I: control (treated with normal saline); Group II: 8mg/kg diquat (chronic mild stress, CMS); Group III: (12mg/kg diquat (chronic moderate stress, COS). The animals were injected intraperitoneally with normal saline or diquat (8 or 12mg/kg), twice a week for four consecutive weeks. Diquat was dissolved in normal saline to a concentration of 2 mg/ml (pH 7.4) and filter-sterilised. Control animals received the same volume of normal saline alone. For the *in vivo* experiments, we chose to use diquat at the doses of 8~12 mg/kg because previous reports have indicated that the acute toxicity is induced by an intraperitoneal injection of 24 mg/kg body weight[3]. Thus, we used 8~12 mg/kg as the optimum dose for 4 weeks to model the chronic oxidative stress in female mice. All mice were anesthetized with halothane (3% for induction and 1.5% for maintenance) in 30% oxygen and 70% nitrous oxide using a face mask. These mice were sacrificed by cervical dislocation in a state of unconsciousness induced by inhalation, and then the ovaries were removed. Throughout the course of this study, animals treated with diquat or normal saline did not die prior to the experimental endpoint. After obtaining the weight of the mice, blood samples were collected from orbital venous plexus for biochemical assays. The ovaries were prepared immediately for further examinations. Granulosa cells were isolated from the left ovaries for ROS level measurement, and the right ovaries were fixed in 4% paraformaldehyde for the terminal deoxynucleotidyl transferase dUTP nick-end labeling (TUNEL). Germinal vesicle (GV) oocytes were harvested from the ovaries of control and diquat-treated mice and cultured in M16 medium under paraffin oil at 37°C in a 5% CO_2_ atmosphere. After culture, the oocytes were harvested for developmental competence measurement.

### Determination of ROS generation

To determine the quantity of ROS production, follicular granulosa cells and oocytes were collected by puncture of the dominant ovarian follicle from the ovaries of control and diquat-treated ICR mice. ROS levels in granulosa cells and oocytes were measured using the intracellular ROS red fluorescence determination kit. All procedures were performed according to manufacturer's instructions. This assay is based on the principle that dihydroethidium bromide (DHE) can pass through cell membrane to enter into cells and be oxidized into ethidium bromide by intracellular ROS, which produces a red fluorescent signal when binds to DNA in the nucleus. Then, the cells were washed twice with PBS and stained with 4',6-diamidino-2-phenylindole (DAPI) for the identification of nucleus. Images were taken using a laser-scanning confocal microscope (Leica, Wetzlar, Germany) and analyzed with ImageJ 1.42q software. A region was defined for granulosa cells or oocytes, and the average fluorescence intensity value of per granulosa cell or oocyte within the region was measured. The average fluorescence intensity values from all measurements were obtained and used for statistical analysis. Each experiment was repeated at least three times and at least 200 granulosa cells and 30 oocytes were examined for each group.

### TUNEL assay

The right ovaries from control and diaquat-treated mice were fixed in 4% paraformaldehyde for the TUNEL assay. The procedure was performed according to the in situ cell death detection kit protocol. Briefly, the right ovaries embedded in paraffin were cut every 5~7μm and sections were placed onto glass slides. After deparaffinization and rehydration, the sections were incubated with 250 mg/ml proteinase-K for 15 min at 37°C and rinsed with PBS for 15 min. Then, the sections were incubated in a humidified chamber with 0.1% Triton 100 X for 10 min at 4°C, washed for 10 min with phosphate-buffered saline (PBS), and terminal deoxynucleotidyl transferase stained for 70 min at 37°C, and stained with DAPI. Images were obtained using a laser scanning confocal microscope. Follicles were considered atretic if encircled by five or more TUNEL-positive ovarian granulosa cells[23]. Cells were defined as TUNEL-positive if they stained bright green in the nuclei with the TUNEL assay. Total follicles and TUNEL-positive follicles were quantified in sections taken at the maximum diameter of each ovary.

### Biochemical assay

Blood samples were centrifuged at 4000 rpm for 10 min. Serum was collected to determine biochemical parameters. The activities of T-SOD, CAT, GPx, and MDA content were measured using commercial reagent kits (Nanjing, China). Analyses of T-SOD activity was based on SOD-mediated inhibition of nitrite formation from hydroxyammonium in the presence of O_2_^–^ generators (xanthine/xanthine oxidase)[24]. GPx activity was estimated by the analysis of reduced GSH in the enzymatic reaction[25]. CAT activity was measured by analyzing the rate at which it caused the decomposition of H_2_O_2_ at 240 nm[26].

### Quantitative real-time polymerase chain reaction (qPCR)

Total RNA was extracted from granulosa cells using TRIzol reagent (Invitrogen, Carlsbad, CA, USA) according to the manufacturer protocol. First cDNA strand was synthesized using PrimeScript™ RT Master Mix (Takara). Quantitative real-time PCR (qRT-PCR) was conducted using a fast real-time PCR system (Roche LightCycler® 480 system). Triplicate samples were assessed for each gene of interest, and GAPDH was used as a control gene. Relative expression levels were determined by the 2^−ΔΔCt^ method. Sequences of primers used for apoptosis related genes are listed in Table 1.

**Table 1:** Primer sequences for real-time RT-PCR.

Gene	Forward Primer	Reverse Primer	Base pairs	T	Accession Number
GAPDH	ATGGTGAAGGTCGGTGTGAACG	CTCGCTCCTGGAAGATGGTGATG	452	56	NM_008084.2
Bax	CCAGGATGCGTCCACCAAGA	GGTGAGGACTCCAGCCACAA	394	57	NM_007527.3
Bcl-2	GTGGATGACTGAGTACCTGAACC	AGCCAGGAGAAATCAAACAGAG	120	60	NM_009741.3
Bim	TATGGAGAAGGCATTGAC	TGTGGTGATGAACAGAGG	207	56	NM_009754.3
Caspase-3	ACAGCACCTGGTTACTATTC	CAGTTCTTTCGTGAGCAT	255	54	NM_009810.2

Abbreviations: GAPDH, glyceraldehyde-3-phosphate dehydrogenase; Bax, Bcl-2 associated X protein; Bcl-2, B-cell leukemia/lymphoma 2; Bim, B-cell lymphoma-2(Bcl-2)-interacting modulator of cell death; Caspase-3, Cysteinyl aspartate-specific proteinase-3; T, the annealing temperature given as°C.

### Reproductive performance

After normal saline or diquat treatment for four weeks, all mice were then paired with male mice (1:1) overnight. Vaginal plug were examined in the following morning, and the presence of a vaginal plug was determined as gestation 0.5 day (GD 0.5). The number of fetal mice and average litter size were counted.

### Statistical analysis

Statistical analysis was performed using the SPSS 16.0 software (SPSS Inc., Chicago, IL, USA). All values are expressed as mean ± S.E.M. The statistical significance between groups was analyzed by one-way ANOVA and a P value of < 0.05 was considered significant. All experiments were repeated at least three times.

## Results

### Effect of diquat on ovary weight and oocyte developmental competence

**Fig 1:**
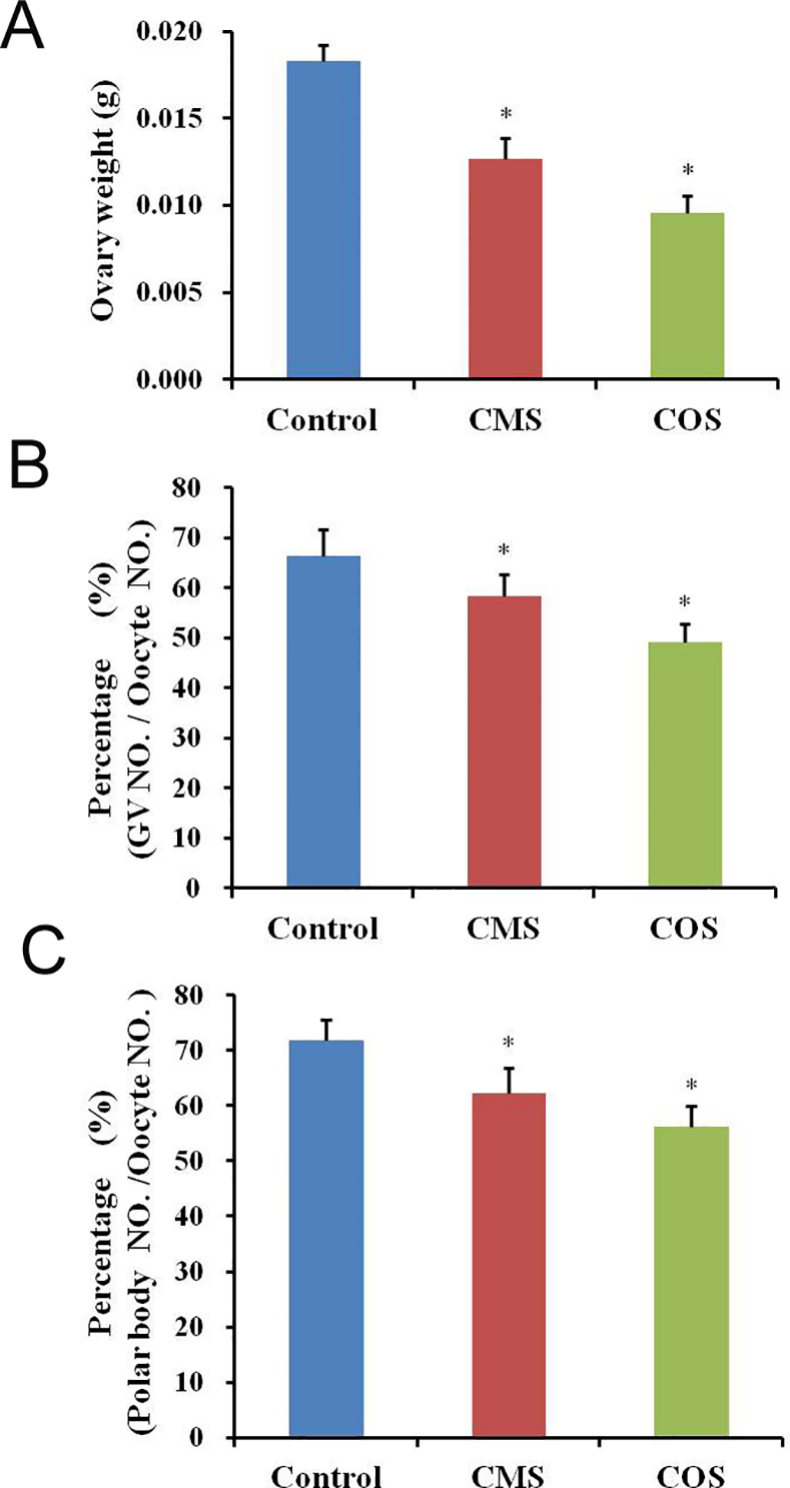
Effect of diquat on the ovary weight and oocyte quality. (A) In diquat-treated mice, ovary weights were significantly reduced as compared to those of control mice. (B) The percentage of normal GV oocytes was significantly decreased after diquat treatment. (C) In diquat-treated mice, the percentage of polar body extrusion was significantly reduced after culture for 12h. Results given are mean ± SEM, n = 6. Asterisk (*) indicates significant differences versus control (*P* < 0.05).

To investigate the effect of diquat-induced damage on female reproductive organs, we measured variations in the ovary weight. In the diquat-treated mice, ovary weights (CMS:0.0127±0.00116g; COS:0.0096±0.00097g) were significantly reduced compared with those in control mice (0.0183±0.00092g; Fig 1A). Moreover, the percentage of normal GV oocytes was significantly decreased after diquat treatment (CMS: 58.3±4.3%, n = 196; COS: 49.1±3.7%, n = 207) than that from control mice (66.4±5.2%, n = 205; *p*<0.05; Fig 1B). Next, we assessed the effect of diquat on the percentage of polar body extrusion in oocytes. The oocytes were collected from ovaries and cultured for 12 h. The percentage of polar body extrusion was significantly reduced after diquat treatment (CMS: 62.2±4.6%, n = 112; COS: 56.3±3.8%, n = 106) as compared to that from control mice (71.8±3.9%, n = 214, *p*<0.05; Fig 1C).

### Diquat causes oxidative stress in follicular granulosa cells and oocytes

**Fig 2:**
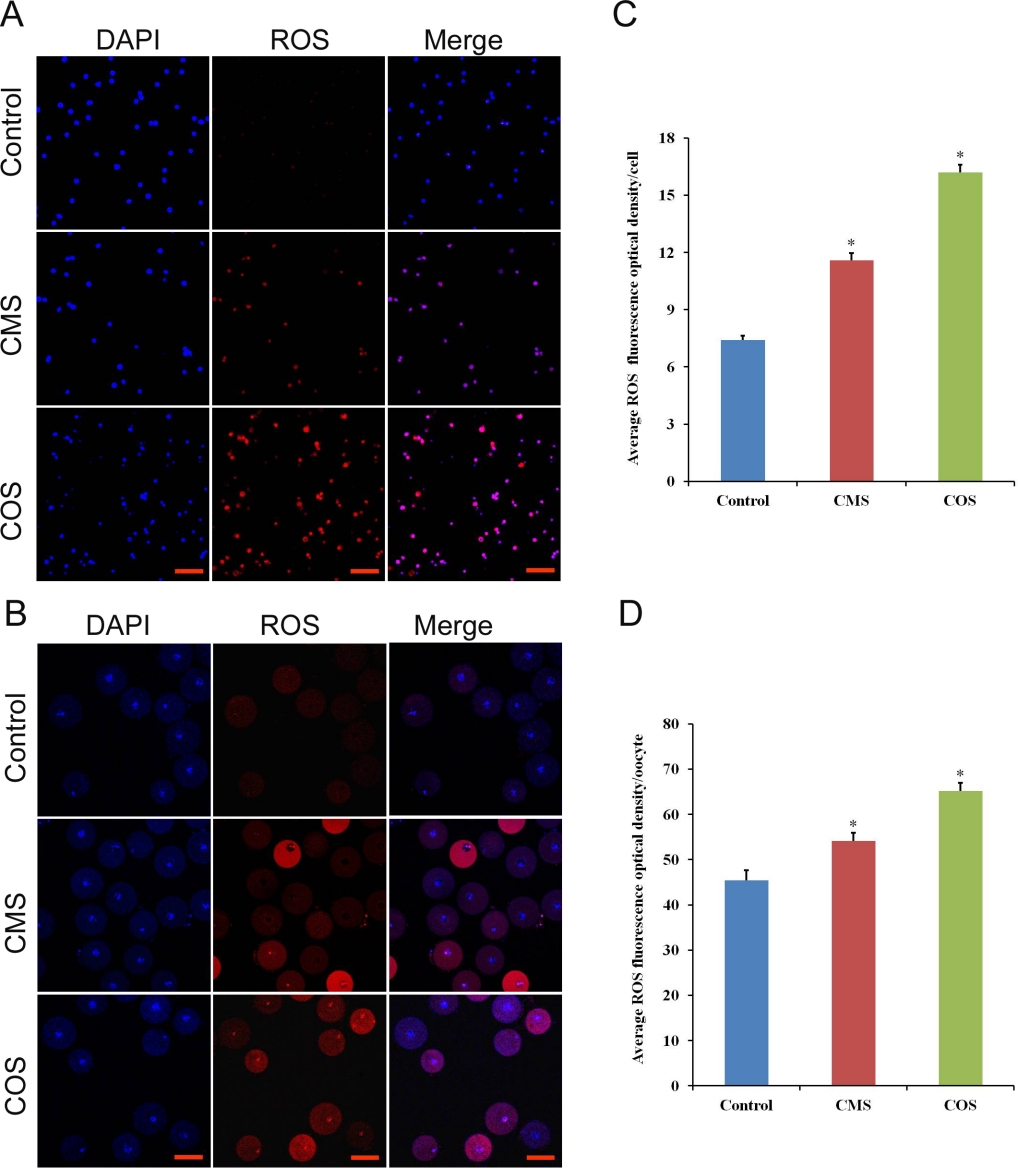
Effect of diquat on ROS generation in granulosa cells and oocytes. (A and B) Fluorescence (red) ROS levels in follicular granulosa cells and oocytes were determined by dihydroethidium bromide. Scale bars are 100μm, DAPI (blue). (C and D) Relative fluorescence intensity in each cell was analyzed by ImageJ software. Data are presented as mean ± SEM, *significant difference from control (*P* < 0.05).

To further evaluate the effect of diquat on follicular granulosa cells and oocytes, we examined intracellular ROS production. As shown in S1 Fig, the ROS production in follicular granulosa cells were significantly increased when chronic exposed to diquat. Furthermore, ROS red fluorescence intensity of follicular granulosa cells and oocytes were significantly increased after diquat treatment (granulosa cell, CMS:11.6±0.36, n = 200; COS:16.2±0.41, n = 200), (oocyte, CMS:54.1±2.23, n = 56; COS:65.2±1.8,n = 1.8) as compared to those in control treatment (granulsoa cell, 7.4±0.23, n = 200, *p*<0.05; Fig 2C), (oocyte, 45.4±2.23, n = 60, *p*<0.05; Fig 2D), which indicated that diquat caused oxidative stress in follicular granulose cells and oocytes.

### Diquat induces follicular granulosa cell apoptosis

**Fig 3:**
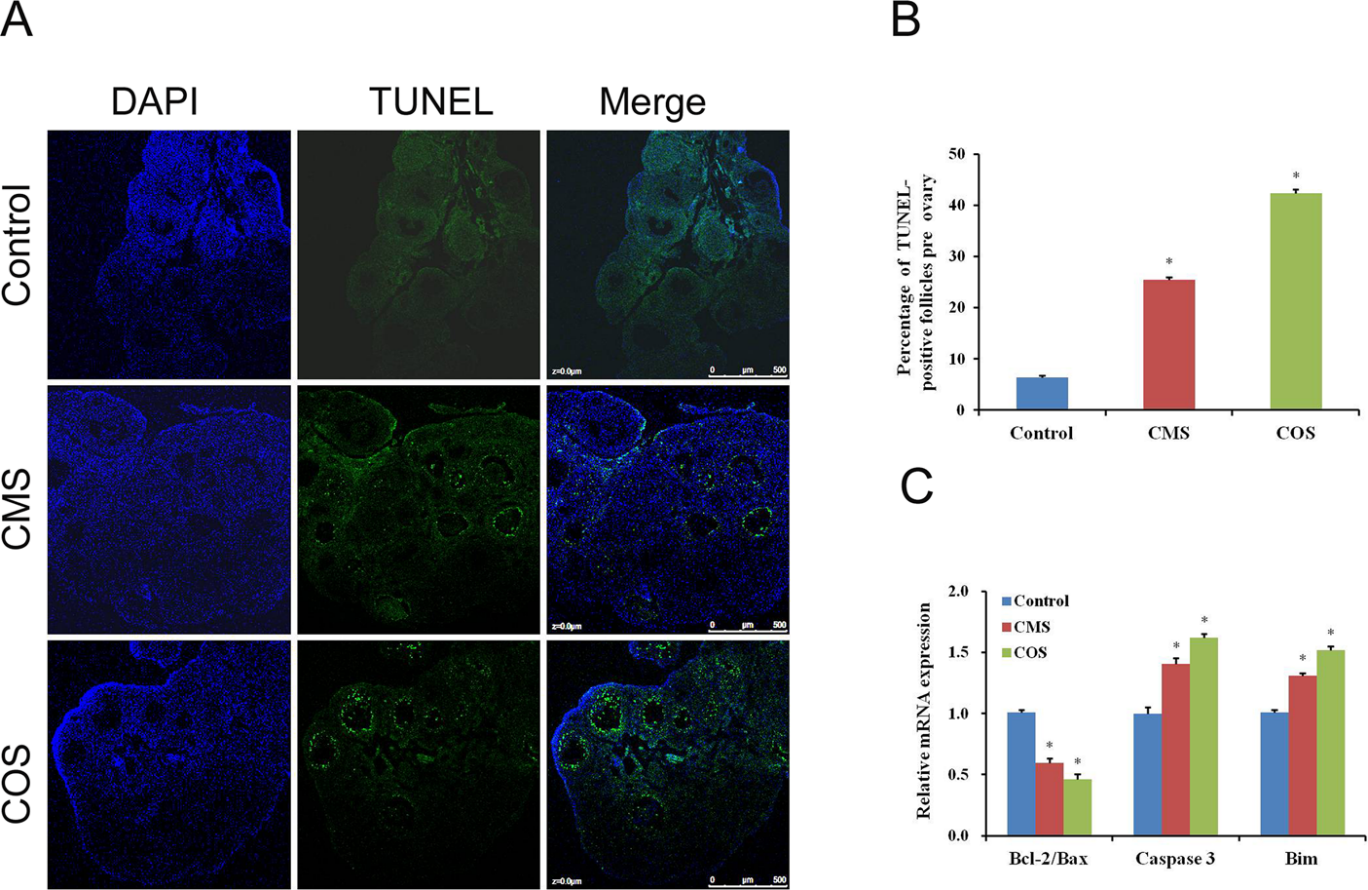
Effect of diquat on follicular granulosa cell apoptosis. (A) TUNEL assay of follicular granulosa cells in ovarian sections. Scale bars are 100μm, n = 6. (B) Quantification of TUNEL-positive follicles. (C) The relative mRNA levels of apoptosis-related genes in granulosa cells. Values represent mean ± SEM. *Significantly different from control (*P* < 0.05).

Apoptosis plays an important role in determining cell fate. Thus, we assessed apoptosis occurrence in follicular granulosa cells. TUNEL staining was applied to identify the number of apoptotic granulosa cells (TUNEL-positive) and follicles were defined as atretic if encircled by five or more TUNEL-positive granulosa cells [23]. Cells were identified as positive if they stained bright green in the nuclei with the TUNEL assay. As shown in Fig 3A, TUNEL staining results showed that follicular granulosa cells exhibit significantly higher apoptosis rates in ovarian sections from diquat-treated mice as compared to those of control mice. Furthermore, the percentage of TUNEL-positive follicles was significantly higher (CMS: 17.5±0.45%, n = 132; COS: 26.3±0.82%, n = 126) in diquat treated mice than that of controls (6.4±0.35%, n = 154, *p*<0.05; Fig 3B). RT-PCR results also showed that the mRNA expression levels of pro-apoptotic genes (Bim and caspase-3) significantly increased (CMS: 1.41±0.043 and 1.31±0.023; COS: 1.62±0.056 and 1.52±0.021 vs. 1.0; *p* < 0.05) and the ratio of Bcl-2 to Bax decreased (CMS: 0.60±0.043; COS: 0.46±0.031 vs. 1.0; *p* < 0.05) in granulosa cells from diquat treated mice than those from control mice (Fig 3C).

### Effect of diquat on activities of antioxidant enzymes and MDA content in the serum

**Fig 4:**
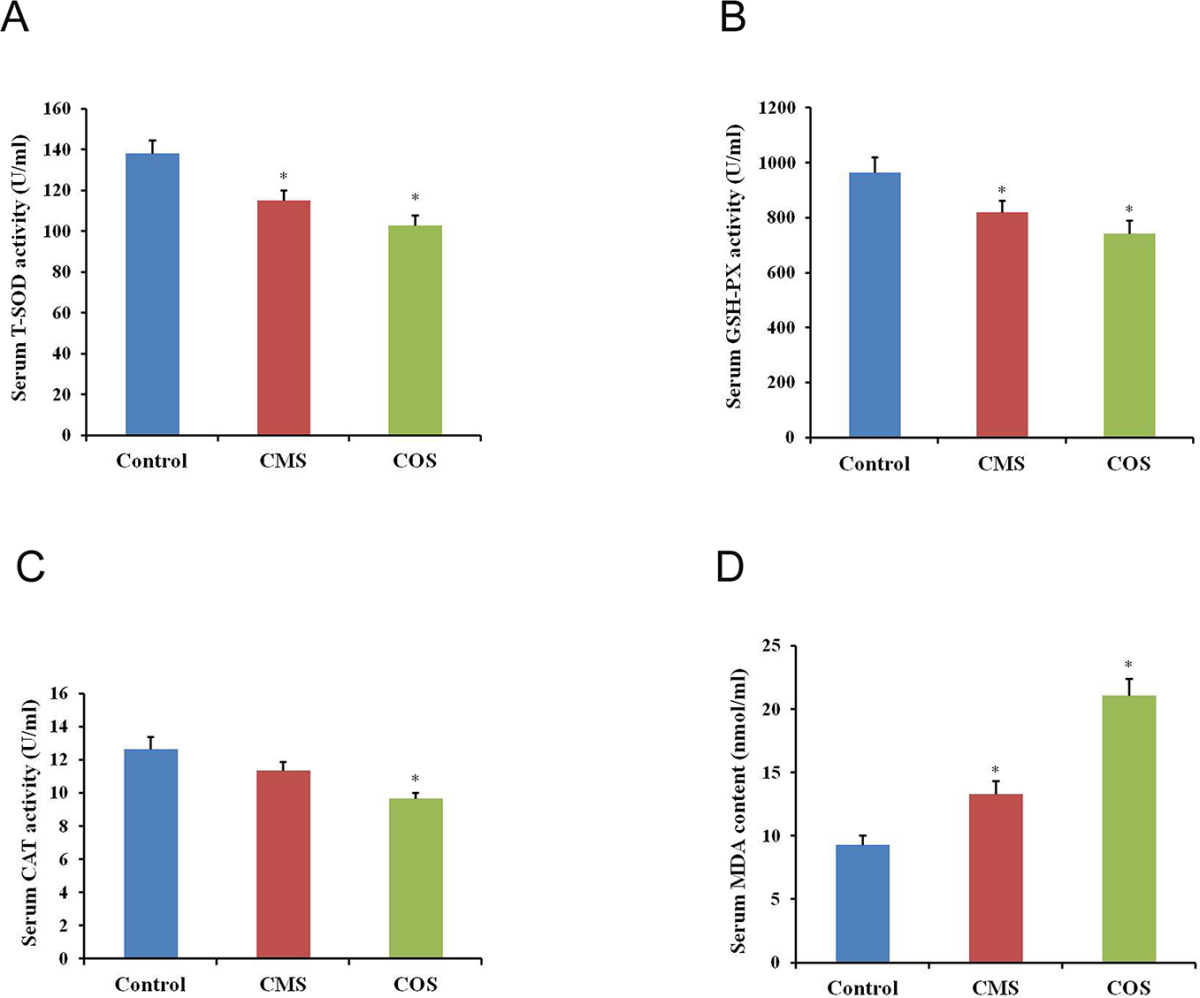
Effect of diquat on serum antioxidant enzymes activities and MDA content. (A) Activity of T-SOD in mouse serum. (B) Activity of GSH-Px in mouse serum. (C) Activity of CAT in mouse serum. (D) Content of MDA in mouse serum. Results given are mean ± SEM, n = 6. Asterisk (*) indicates significant differences versus control (*P* < 0.05).

The activities of antioxidant enzymes and MDA content were sensitive indicators for oxidation stress. Antioxidant enzymes can directly detoxify ROS. SOD can react with superoxide anion radicals to form the less dangerous H2O2, which is further degraded by CAT and GSH peroxidases (GPXs) to water[27]. Thus, we examined the activities of T-SOD, CAT and GSH-Px as well as the MDA content in the serum. The results showed that the activities of antioxidant enzymes (T-SOD, GPx and CAT) decreased significantly (CMS:115.04±5.25,812.06±42.31 and 11.35±0.53; COS: 103.08±4.84,742.26±48.05 and 9.67±0.34) in the serum from diquat-treated mice as compared to that of the control group (138.24±6.61, 965.07±56.13 and 12.64±0.74, *p*<0.05; Fig 4A–4C). Additionally, the MDA content increased significantly (CMS:13.03±0.75; COS: 21.08±0.35) after diquat treatment than that from control mice (9.28±0.75, *p*<0.05; Fig 4D).

### Effect of exposure to diquat on fetus development and litter size

**Fig 5:**
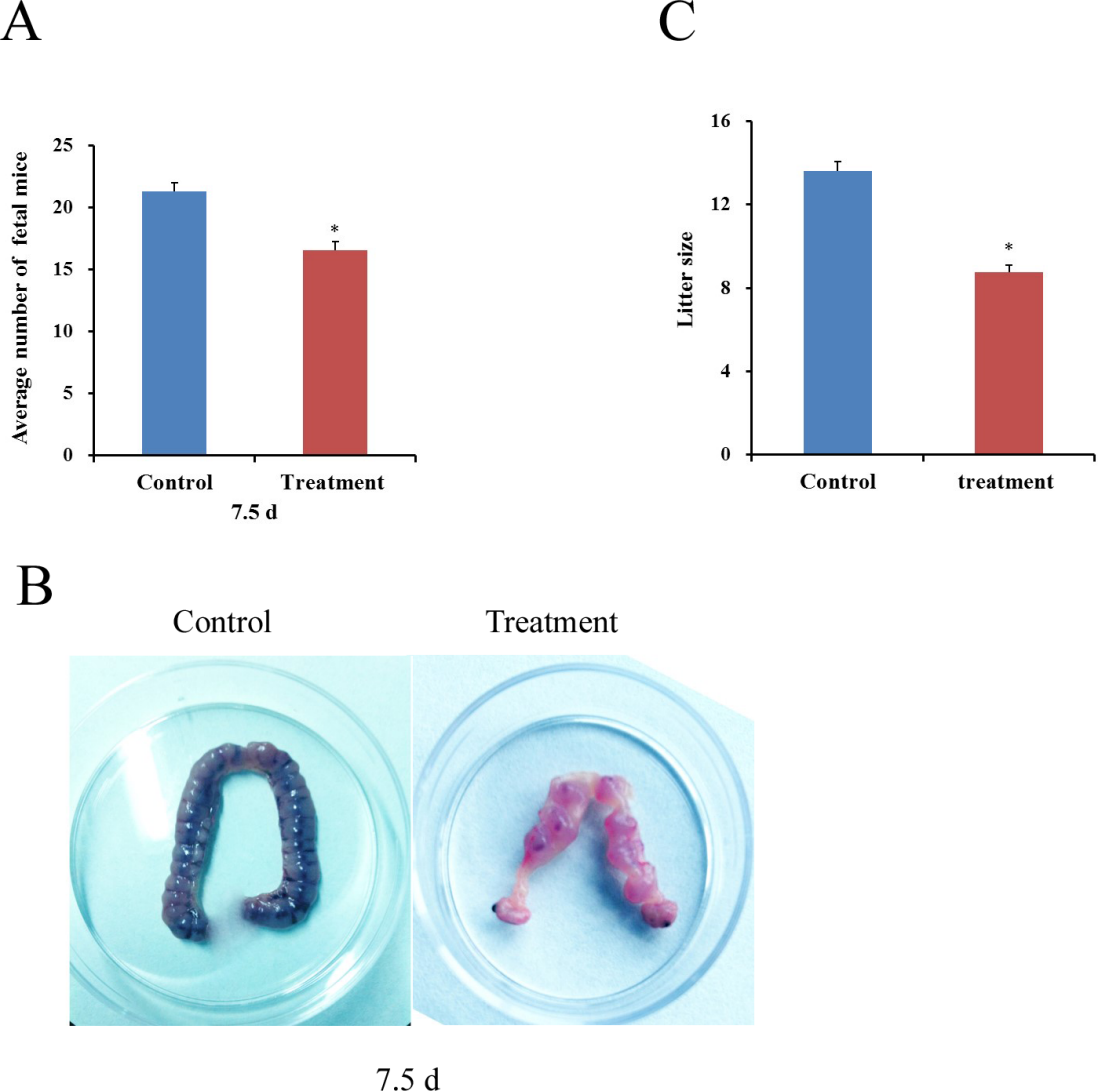
Effect of diquat on the numbers of fetus and litter size. Female mice were intraperitoneally injected with saline or diquat for 4 weeks, respectively. (A and B) Diquat effects on fetus development in vivo. (C) Diquat effects on mouse litter size Values represent mean ± SEM, n = 10. *Significantly different from control (*P* < 0.05).

To further assess the effect of diquat on female mice reproductive toxicity, we examined the numbers of fetus on GD 7.5d and offspring after diquat treatment. The average numbers of fetus on GD 7.5d and litter size were significantly reduced after diquat treatment (16.56±0.74 and 8.75±0.34) compared with those of control group (21.32±0.67 and 13.65±0.47; *p*<0.05; Fig 5A and 5C). Moreover, at the same gestational stages (GD7.5d), the diquat treatment group retarded fetus development (Fig 5B).

## Discussion

In this study, we used diquat to induce oxidative damage to evaluate the reproductive toxicity of it in female mice, simulating the toxic effects of human and animal chronic exposure. We examined the toxic effects of diquat on oocyte development competence, ROS levels in granulosa cells and oocytes, granulosa cell apoptosis, early embryo development in vivo, and litter sizes. We found that chronic exposure to diquat had adverse effects on oocyte quality, follicular development, fetus development and litter sizes. Further, our data indicated that chronic exposure to diquat decreased activities of T-SOD, GSH-Px, and CAT and increased the levels of MDA in the serum.

Previous studies have reported its toxicity in the liver[4, 28], kidney[12], and small intestine[29]. In addition, diquat induced oxidative stress and cytotoxicity in vitro cultured cells [6].However, there is little information regarding the effect of chronic exposure to diquat on the female mammalian reproductive system, particularly ovarian function. In this study, we for the first time showed that chronic exposure to diquat reduced oocyte quality, impacted early embryo development and litter sizes. These results were consistent with those in a previous report that showed that diquat-treated mallard embryos resulted in malformations and oxidative stress in hatchlings[30]. In the present study, we focused on the effect of diquat on the quality of oocytes, since oocyte quality is critical for oocyte maturation, fertilization, and embryo quality[31]. Our results showed that the percentage of GV oocytes and polar body extrusion was significantly reduced in diquat-treated mice as compared to that of the control mice. These results indicated that chronic exposure to diquat had a toxic effect on oocyte developmental competence.

ROS are usually formed in the process of oxidative phosphorylation and ATP production and include superoxide anion, hydrogen peroxide, hydroxyl radical, singlet oxygen, and others [32]. In addition, P450 enzymes within ovarian tissues are also sources of ROS[27]. Physiological levels of ROS are required for normal cell function, energy production, intercellular signalling regulation, follicle development and ovulation. Oxidative stress occurs when increased ROS levels exceeding their eliminative rate, resulting in adverse effects to cellular macromolecules [33]. Normal ovarian function is very critical to maintain follicle development, ovulation, fertility and health. Proper ROS levels within follicles are necessary for the normal development of the ovarian follicle and subsequent oocyte and embryo growth[22]. However, excessive ROS levels is detrimental to female reproductive system, particularly, ovarian function[34]. Moreover, oocytes and embryos are extremely sensitive to ROS, and a slightly increased ROS level can reduce oocyte quality, disrupt early embryo development and result in embryo fragmentation[20]. Studies have shown that ROS are involved in the initiation of granulosa cell apoptosis and antral follicular atresia [18, 35]. Furthermore, antral follicles appear to be highly sensitive to oxidative stress-induced apoptosis of granulosa cells[27]. Our results showed that diquat lead to high ROS levels production in oocytes and antral follicular granulosa cells, which was an important factor for depressed oocyte quality and developmental competence and increased TUNEL-positive follicles rates. Previous studies indicated that higher ROS levels could cause oxidative damage in the mouse ovary[16] and result in apoptosis among oocytes and embryos[36]. Our results suggested that diquat increased the ROS levels in oocytes and granulosa cells, which further caused granulosa cell apoptosis and decreased oocyte development competence.

Apoptosis is an important cellular processes that plays major roles in determining cell fates[37]. Diquat has been widely used as pro-oxidant to induce oxidative stress in cultured cells[6] and different animal models[8, 9]. However, there were no studies on chronic exposure to diquat in female animals have focused on ovarian function. It is important to investigate the toxic effects of diquat on ovary because it is an extremely critical organ for reproductive function in female animals. Moreover, oxidative stress has been considered as one of the most key factors leading to apoptosis and follicular atresia[18]. Our results showed that apoptosis mainly occurred in antral follicular granulosa cells in diquat-treated mice, as shown by TUNEL-positive granulosa cells and increased apoptosis-related genes expression, which further indicated the toxic effects of diquat on follicular development.

Cells usually protect themselves from ROS damage through the intracellular antioxidant enzymes, including T-SOD, GSH-Px, CAT and so on. In this study, we specifically evaluated the effect of diquat treatment on the activities of antioxidant enzymes in the serum. SOD activity is known to be important for ovarian function and is responsible for dismutation of superoxide anion radicals to oxygen and hydrogen peroxide[35]. The hydrogen peroxide is then further processed to form nontoxic water by either CAT or GPX. The antioxidant enzymes (GPX and CAT) protect SOD against inactivation by H_2_O_2_, and reciprocally SOD protects GPX and CAT from becoming inactivated by superoxide radicals[16]. In this study, the serum activities of the antioxidative enzymes (T-SOD, GSH-Px, CAT) were significantly decreased and the content of MDA was significantly increased in diquat-treated mice, which indicated that antioxidative capabilities of diquat-treated mice were damaged.

Chronic exposures to pesticides and herbicides have shown to induce oxidative stress and cause reproductive toxicity [16, 38, 39]. Diquat is a bipyridyl herbicide that utilizes molecular oxygen to produce super anion radicals and subsequently hydrogen peroxide[30]. Previous studies have suggested that treatment of animals and cells with diquat provides an ideal model with which to study the effects of oxidative stress[9, 40]. However, there were no studies on chronic exposure to diquat in female reproductive toxicity. Our results indicated that after diquat treatment, the numbers of fetal mice and offspring were significantly reduced, which further demonstrated the toxic effects of diquat on oocyte quality, embryo developmental competence and litter sizes.

In this study, diquat-induced reproductive toxic effects in female mice were investigated. Our results showed that diquat could have effects on the female reproductive system by enhancing ROS levels within oocytes and granulosa cells, inducing granulosa cell apoptosis and follicular atresia, reducing oocyte quality and developmental competence, and affecting fetus development and litter sizes. This study lays the foundation for the research of ovarian oxidative stress and possible mechanisms of action in domestic animals.

## Supporting Information

S1 Fig Effect of chronic exposure to diquat on ROS production in follicular granulosa cells. Intracellular ROS levels were quantified by colorimetric assay. ROS levels were significantly higher in experiment groups than control group. Bars represent means±SEM, n = 3.*significant difference from control (*P* < 0.05).
(TIF)
