# Supplementary figures and images for: Chronic Exposure to Diquat Causes Reproductive Toxicity in Female Mice
Source: PLoS One. 2016 Jan 19;11(1):e0147075. doi: 10.1371/journal.pone.0147075 (PMC4718508; doi:10.1371/journal.pone.0147075)

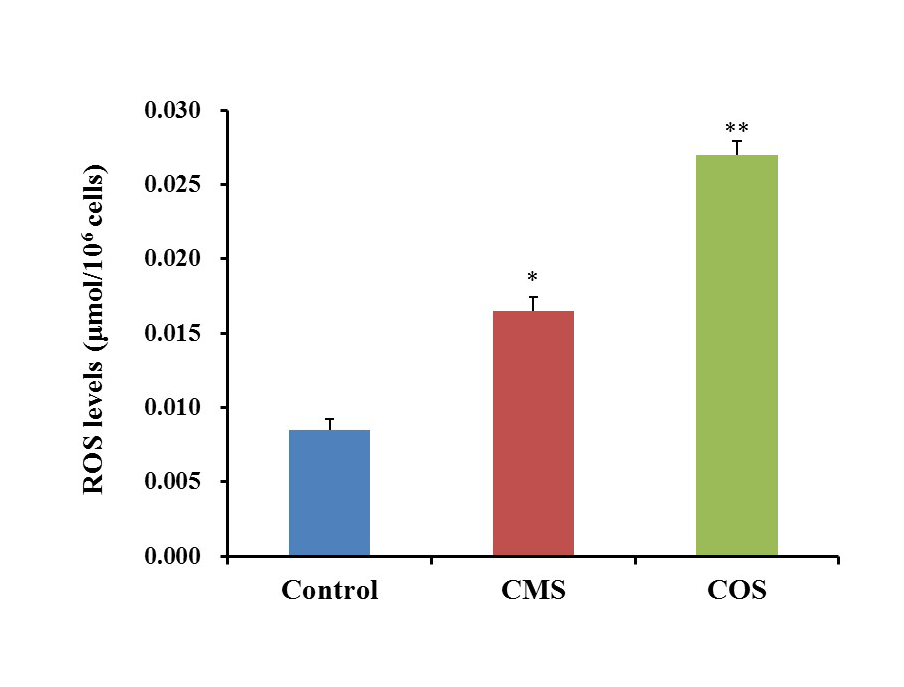

Supplement: S1 Fig — Intracellular ROS levels were quantified by colorimetric assay. ROS levels were significantly higher in experiment groups than control group. Bars represent means±SEM, n = 3.*significant difference from control (P < 0.05). (TIF) [file pone.0147075.s001.tif]
